# Developing a new self-reported scale of oral health outcomes for 5-year-old children (SOHO-5)

**DOI:** 10.1186/1477-7525-10-62

**Published:** 2012-06-07

**Authors:** Georgios Tsakos, Yvonne I Blair, Huda Yusuf, William Wright, Richard G Watt, Lorna M D Macpherson

**Affiliations:** 1 Department of Epidemiology and Public Health, UCL, London, UK; 2 Oral Health Directorate, NHS Greater Glasgow & Clyde, Glasgow, UK; 3 Community Oral Health, Dental School, University of Glasgow, Glasgow, UK

**Keywords:** Outcome measure, Psychometrics, Qualitative research, Quality of life, Validation

## Abstract

****Background**:**

Information on the impact of oral health on quality of life of children younger than 8 years is mostly based on parental reports, as methodological and conceptual challenges have hindered the development of relevant validated self-reported measures. This study aimed to develop and assess the reliability and validity of a new self-reported oral health related quality of life measure, the Scale of Oral Health Outcomes for 5-year-old children (SOHO-5), in the UK.

****Methods**:**

A cross-sectional study of two phases. First, consultation focus groups (CFGs) with parents of 5-year-olds and review by experts informed the development of the SOHO-5 questionnaire. The second phase assessed its reliability and validity on a sample of grade 1 (5-year-old) primary schoolchildren in the Greater Glasgow and Clyde area, Scotland. Data were linked to available clinical oral health information and analysis involved associations of SOHO-5 with subjective and clinical outcomes.

****Results**:**

CFGs identified eating, drinking, appearance, sleeping, smiling, and socialising as the key oral impacts at this age. 332 children participated in the main study and for 296 (55% girls, mean d_3_mft: 1.3) clinical data were available. Overall, 49.0% reported at least one oral impact on their daily life. The most prevalent impacts were difficulty eating (28.7%), difficulty sleeping (18.5%), avoiding smiling due to toothache (14.9%) and avoiding smiling due to appearance (12.5%). The questionnaire was quick to administer, with very good comprehension levels. Cronbach’s alpha was 0.74 and item-total correlation coefficients ranged between 0.30 and 0.60, demonstrating the internal consistency of the new measure. For validity, SOHO-5 scores were significantly associated with different subjective oral health outcomes (current toothache, toothache lifetime experience, satisfaction with teeth, presence of oral cavities) and an aggregate measure of clinical and subjective oral health outcomes. The new measure also discriminated between different clinical groups in relation to active caries, pulp involvement, and dental sepsis.

****Conclusions**:**

This is the first study to develop and validate a self-reported oral health related quality of life measure for 5-year-old children. Initial reliability and validity findings were very satisfactory. SOHO-5 can be a useful tool in clinical studies and public health programs.

## **Background**

Despite general improvements in oral health, dental caries is a major public health problem affecting 60%–90% of children globally [1,2]. Consequently, children are the focus for oral health policy in many countries [3,4] and measurement of oral health and quality of life should be key outcomes for evaluating oral health programs. Dental conditions, untreated dental caries in particular, have been linked with delayed growth and cognitive development of the child [5-9]. Furthermore, they can negatively impact on the daily life of children and affect their families psychologically and economically [8,10-13].

Many “oral health related quality of life” (OHRQoL) measures have been developed and tested in various populations to assess the impacts of oral conditions on the daily life of people [14]. Such measures have also been developed specifically for child and adolescent populations, in line with the respective general health measures [15-18]. However, they have predominantly focused on children aged 8 years and older [19-22], with the exception of a parental report measure for children aged 6 years and older [12]. While a self-reported set of OHRQoL questions has been used on very young children [23], those were treated as independent questions, rather than a composite measure, without presenting concrete evidence on psychometric properties. Recently, an OHRQoL measure that included both parental and children reports was developed and validated for preschool and school-aged children, though the child self-report version was only used on children aged 8 years or older [24].

The relatively limited research in this area is a direct consequence of methodological and conceptual challenges for developing OHRQoL measures for very young children. Children’s perceptions about the impact of oral conditions on their life are based on their experience of oral diseases and are influenced by their immediate family environments and the wider social context including friends, schools and neighbourhoods [16,25,26]. Furthermore, their understanding of illness and health is age dependent due to social, language, emotional, and cognitive development [16,26]; these developmental stages should be carefully considered within the appropriate social contexts when constructing subjective measures for young children [27]. Abstract thinking is not initiated before the age of 6 years and understanding of even basic health concepts may be problematic in younger children [28]. It is even later in childhood that they can evaluate their feelings and thoughts and compare them with those of their peers [29]. All these challenges have led to the lack of appropriate measures or the use of parental reports as proxies for young children’s perceptions of OHRQoL. On the other hand, there is evidence that 4–6 year-old children can report reliably on more concrete domains of their own general health and quality of life, including pain and dysfunction, though not on abstract domains such as emotional well-being [30]. However, using general health related quality of life measures would be useful but insufficient for measurement of quality of life aspects related specifically to oral health, as has also been shown in adult populations [31,32].

Hence, it is important to develop and validate appropriate self-reported OHRQoL measures for very young children. This study aimed to develop and assess the reliability and validity of a new self-reported OHRQoL measure, the Scale of Oral Health Outcomes for 5-year-old children (SOHO-5), in the UK.

## **Methods**

This cross-sectional study comprised of two phases. The first phase included consultation focus groups (CFGs) with parents of 5-year-olds. This informed the development of the questionnaires used in the second phase on 5-year-old children. Both phases were carried out in the Greater Glasgow and Clyde National Health Service (NHSGGC) Board area in the west of Scotland. The study was undertaken as part of the Childsmile program evaluation strategy. Childsmile is a program in Scotland designed to improve the children’s oral health and reduce inequalities in dental health and access to dental services [33]. Ethical approval was obtained from the West Glasgow Ethics Committee. All parents in the CFGs and parents of all children that participated in the second phase provided written consent.

### **Qualitative phase - development**

CFGs with parents or legal guardians of grade 1 primary (5-year-old) schoolchildren were arranged in schools stratified by socioeconomic status (SES) into affluent, middle, and deprived using the DepCat index, a composite socio-economic position measure based on census data [34] that was widely used in Scotland for several decades. Overall, 25 parents provided positive consent for their participation in the CFGs and 8 of them attended the discussions; we carried out three CFGs, one for each socioeconomic stratum. The participants of the three CFGs were informed that the broader study aimed to develop and test an OHRQoL questionnaire for young children and their parents and that the CFGs were the first step in this process. A detailed information sheet provided an outline of the CFGs discussion points.

The CFGs discussions initially focused on broader concepts and concerns in relation to oral health, such as exploring parental views about the importance and key determinants of their children’s oral health. Then, we moved into common oral health problems for a 5-year-old, as well as impacts of oral conditions on children’s and family lives. This provided input in relation to specific items that should be included in OHRQoL questionnaires for children aged 5 years; the rating scales; the questionnaire’s content and structure; and the appropriateness of the language. Based on review of previous work in this field, in terms of both composite OHRQoL measures and more simple inventories, some potential questions were further presented for discussion. In addition, potential questions for inclusion in the new measure were also reviewed by a number of experts in pediatric dentistry, public health, and health related quality of life, including consultants and academics in dental public health, health services researchers, child dental services programme managers and the Chief Dental Officer in Scotland. The experts, two of which are members of the Childsmile program Evaluation Board, reviewed the potential questions and suggested other questions independently.

This process facilitated the selection of relevant questions, adaptation of the language to reflect regional idiom, and informed the structure and content of the draft questionnaire to be tested in the field. The questionnaire consisted of an initial section on toothache experience and current perceptions, followed by questions assessing oral impacts on the usual daily activities of a child of that age. These latter questions formed the SOHO-5. Children were asked whether they had experienced any difficulties with: eating, drinking, speaking, playing, smiling (because teeth hurt), smiling (because of the way teeth look), and sleeping. The questions were worded simply (e.g. Has it ever been hard for you to eat because of your teeth?) and the answer consisted of 3 options (no; a little; a lot) facilitated by a prompt/explanation card with relevant faces, in line with the consistent recommendation of all three CFGs. The SOHO-5 questionnaire and prompt/explanation cards can be provided on request.

### **Quantitative phase – validation**

The sample was derived from schools included in the National Dental Inspection Programme (NDIP). The NDIP offers an annual basic dental inspection to all grade 1 (5-year-old) and grade 7 (11-year-old) state primary schoolchildren in Scotland. In addition, a more detailed clinical dental examination, using the British Association for the Study of Community Dentistry (BASCD) standardised criteria for caries diagnosis at the D3/d3 level, is carried out on a sample of either grade 1 or grade 7 children (alternates every year). All examiners and recorders are trained and calibrated annually for the age group of study. The NDIP clinical dental examinations for grade 1 children were undertaken over a four month period spanning terms one and two of the academic year, while we separately carried out OHRQoL questionnaire data collection for this study in the third term. Thus, caries epidemiology data was available for record linkage to this study’s OHRQoL data.

Schools in the selected areas in NHSGGC were stratified by SES into affluent, middle, and deprived using the DepCat index. Within each stratum random samples of grade 1 year-groups were selected. For a validation study, convenience samples are sufficient and a sample size of 50 to 200 children has been recommended [35]. To ensure representation of different socioeconomic position groups, the target sample was increased to 300 children, consisting of approximately 100 children in each SES stratum. Overall, 35 schools were selected to achieve the target sample: 6 in the affluent, 12 in the middle and 17 in the deprived group. Oversampling in the middle and deprived SES strata accounted for the expected lower participation rates in these schools.

The children’s questionnaire was interviewer-administered by four trained interviewers. The interview consisted primarily of the SOHO-5 questions. In addition, children were also interviewed about toothache experience, current toothache, satisfaction with teeth, self-rated oral health, and whether they had any cavities (“holes in teeth”). These questions were used to assess the validity of the new measure. All children were able to respond to the questions.

### **Data analysis**

The CFGs discussions were tape recorded and then transcribed. We used thematic analysis of the CFG transcripts in order to identify the key themes that emerged from these discussions and classify them in appropriate and meaningful thematic groups [36]. In relation to the analysis of the quantitative phase, we calculated the overall SOHO-5 score by aggregating responses to the 7 items. As answers were coded as 0 (no), 1 (a little), and 2 (a lot), the score ranged from 0 to 14. “Cannot remember” or “do not know” answers were considered as missing data. The aggregate SOHO-5 score was considered as missing if data on two or more items were missing; otherwise the score was the sum of the items with non-missing data.

The psychometric testing of SOHO-5 involved the assessment of internal reliability, and face, content and construct validity. Internal reliability was tested through Cronbach’s alpha coefficient, item-total and inter-item correlations. Face validity was based around the comprehensibility of the questions. For this, we recorded whether questions were repeated or reworded, and used the count of repeated or reworded questions as an indirect quantitative estimate of children’s understanding. Content validity was based on the CFG findings and the expert panel review.

Without an established gold standard measure, the validation process relied heavily on construct validity. We examined the association of the SOHO-5 score with subjective oral health variables (satisfaction with teeth, reported cavities, current toothache, and toothache experience) and a dichotomous aggregate oral health status variable. This was created by combining information from the NDIP clinical examination and the questionnaire to indicate whether the child had any of the following: dental caries, pulp involvement, current toothache, or toothache experience.

In addition, we used data from the children’s NDIP clinical examination (presence of dental caries, pulp involvement, and dental sepsis) to assess the association between SOHO-5 and clinical measures, thereby looking at its ability to distinguish between different clinical groups. For all aforementioned associations, Mann–Whitney tests were used because the SOHO-5 score was not normally distributed.

## **Results**

### **Qualitative phase - development**

CFGs provided useful insight on the concerns of 5 year-olds’ parents about the oral health of their children and identified common problems related to their dental health. Parents discussed their children’s health related behaviours, mostly in relation to sugar consumption and choosing appropriate toothpaste. There was variation in the parental views in relation to the experiences of dental diseases and the concerns about their consequences. Some parents expressed little concern whereas others reported serious concerns about the consequences of dental disease on their children’s quality of life. The impact of tooth decay and trauma in front teeth in relation to appearance was very prominent:

"“My daughter fell and her front tooth is kind of grey…when it falls out it may affect the next one. That is my main worry because the focus is your face, isn’t it?”"

Parents suggested that as their children got older, their peers were more likely to notice the poor appearance of their teeth and “point at it” which may have social consequences including lack of confidence:

"“My son ended up conscious of that, it affected him for years and his second teeth, it distressed him. As children get older they start asking him what happened to your tooth. It affected the way he smiled. Children start to point. That is when they start worrying about it.”"

Other parents reported that pain was one of the consequences of tooth decay that had considerable impacts on different aspects of their children’s life, such as eating function but also their mood:

"“they don’t realise they had bad teeth, is only when they get older. If it hurts them, it gets to them”; “If their teeth hurt, you get upset and they get upset”"

There was also some implication that awareness of appearance and dental pain are more prominent as a child gets older. The key themes that emerged from the discussions in relation to areas of daily life affected by the impact of oral conditions were eating, drinking, appearance, sleeping, smiling, and socialising.

Finally, CFG participants commented on a draft questionnaire. While broadly satisfied with the content and length, they raised a number of important issues, such as their concern about the 5 year-olds’ concept of time and the usefulness of prompt cards, and suggested language modifications in order to make it more relevant for the local dialect (Table 1). These comments, together with those provided by the expert panel, were used to modify the questionnaire for the main study.

**Table 1:** Summary of Consultation Focus Groups: comments on draft questionnaire

**Questionnaire characteristics**	**Comments**
Content	“Appropriate questions”
	“All main issues covered”
Length	“o.k.”
Concept of time	“They have no concept of time at all”
	“… they don’t know what 7 days is. Maybe a calendar type thing, like Easter, may be useful”
	“..no comprehension of 4 weeks”
Language/vocabulary	“We don’t use the word “pain” or “ache”. I would say either “hurt” or “sore” instead”
	“My children don’t take “hot drinks”. Milk is warm but never hot”
Prompts/cards	“The smiley faces is a good system. They also use them in the nursery. Simpler faces are ok; 5 faces may be too much for a 5-year-old”
	“Need to remind them it is about their teeth”

### **Quantitative phase – validation**

For the validation study, we sent an invitation letter to parents of all eligible children in the selected schools; 416 parents returned positive consent for the participation of their children in the study. Overall, 332 children (79.8% of those with parental consent) participated in the main study; 6 were excluded due to ineligibility and a further 30 could not be matched to NDIP records, therefore 296 children, 134 boys and 162 girls, were included in analyses. Sixty eight percent had no experience of dental decay and the mean overall d_3_mft score was 1.3 (95%CI: 1.0, 1.5). Compared to the respective figures for the broader NDIP population of 5-year-olds in NHSGGC (55% had no experience of dental decay; d_3_mft = 2.16), the dental health of the sample was considerably better.

On average the questionnaire took approximately 5–6 minutes to administer. In relation to comprehension, 82.4% answered the SOHO-5 questions without any repetition or rewording, in 9.5% of the sample the interviewer repeated or reworded a question once, with 8.1% needing more repetitions or rewordings. The two questions on avoiding smiling were the ones that required relatively more repetitions (8.1% for avoiding smiling due to toothache and 7.1% for the same due to appearance).

Overall, 49.0% of participants reported at least one oral impact on their daily life. The most prevalent impact was difficulty eating (28.7%), followed by difficulty sleeping (18.5%), avoiding smiling due to toothache (14.9%) and avoiding smiling due to appearance (12.5%), with the remaining items having lower prevalence (Table 2). The SOHO-5 score ranged between 0 and 14, with a mean of 1.38 (SD: 2.09).

**Table 2:** Prevalence (%) of oral impacts by item and overall (N = 296*)

**OHRQoL questions**	**No**	**A little**	**A lot**
Has it ever been hard for you to **eat** because of your teeth?	71.3	18.9	9.8
Has it ever been hard for you to **drink** because of your teeth?	89.5	7.8	2.7
Has it ever been hard for you to **speak** because of your teeth?	91.5	5.1	3.4
Has it ever been hard for you to **play** because of your teeth?	89.6	8.4	2.0
Have you ever not **smiled** because your teeth were **hurting**?	85.1	9.5	5.4
Have you ever not **smiled** because of how your teeth **look**?	87.5	9.5	3.0
Has it ever been hard for you to **sleep** because of your teeth?	81.5	11.1	7.4
**Oral impact (any of the above)**	**51.0**	**49.0**	

*Bases ranged between 293 and 296 due to missing data.

In terms of internal consistency reliability, inter-item correlation coefficients ranged between 0.11 and 0.44 and the mean was 0.29. Item-total correlation coefficients ranged between 0.30 and 0.60, Cronbach’s alpha coefficient was 0.74 and was lower when any of the items was deleted (Table 3).

**Table 3:** Internal consistency reliability of SOHO-5: item-total correlation coefficients, Cronbach’s Alpha, Alpha if item deleted

	**Corrected item-totalcorrelation coefficients**	**Alpha if item deleted**
Difficulty eating	0.30	0.73
Difficulty drinking	0.42	0.68
Difficulty speaking	0.46	0.69
Difficulty playing	0.60	0.65
Avoiding smiling (due to pain)	0.52	0.65
Avoiding smiling (due to appearance)	0.47	0.67
Difficulty sleeping	0.41	0.68

Cronbach’s Alpha: 0.74

Children that reported current toothache had significantly higher SOHO-5 scores than those without toothache, and the same was the case for toothache experience (Table 4). Furthermore, children that were very satisfied with their teeth had significantly lower SOHO-5 scores, indicating better quality of life, compared to children with lower levels of satisfaction. Similarly, those that did not report having oral cavities had significantly better scores than those that did. Finally, children with poor oral health (any of the following: caries, pulp involvement, current toothache, toothache experience) had worse OHRQoL than those that had none of these conditions. There were also significant differences in OHRQoL between different clinical groups (Table 4). Children with dental caries, those with pulp involvement and those with dental sepsis had significantly worse SOHO-5 scores than children without these conditions.

**Table 4:** Relationship of SOHO-5 score with subjective and clinical oral health indicators (N = 296)

**Oral health indicators**		**N**	**Mean (SD)**	**p**
Toothache (current)	Yes	54	2.39 (2.64)	**<0.001**
	No	242	1.15 (1.88)	
Toothache (experience)*	Yes	102	2.29 (2.62)	**<0.001**
	No	190	0.86 (1.47)	
Satisfaction with oral health	Low	34	1.97 (2.54)	**0.047**
	High	262	1.30 (2.01)	
Reported cavities*	Yes	46	1.72 (1.92)	**0.034**
	No	237	1.27 (2.12)	
Caries	Yes	74	1.69 (2.19)	**0.021**
	No	222	1.27 (2.05)	
Pulp involvement	Yes	31	1.77 (1.61)	**0.010**
	No	265	1.33 (2.13)	
Dental sepsis	Yes	9	3.56 (4.10)	**0.006**
	No	287	1.31 (1.97)	
Poor oral health **	Yes	172	1.85 (2.29)	**<0.001**
	No	124	0.73 (1.55)	

* 4 children in the case of toothache experience and 13 children in the case of reported cavities replied that they “do not know” and were excluded from the analysis.

** Poor oral health is indicated by any of the following: caries, pulp involvement, current toothache, toothache experience.

## **Discussion**

To our knowledge, this is the first study to develop and test a self-reported OHRQoL measure among 5 year-old children. Previous studies on children of this age have mostly used composite OHRQoL measures based on parental reports [12,37]. Despite being commonly used as proxies to assess impacts of chronic conditions on younger children, parents do not always accurately perceive their children’s quality of life, thereby parental proxy reports bring a different perception but do not substitute for children self-reports [38]. This study demonstrated that 5-year-old children are capable of providing their own perceptions of oral impacts and highlighted important challenges in this process. The SOHO-5 was developed in two phases; an initial phase routed on literature review and CFGs informed the generation and selection of the items, while a subsequent quantitative phase provided initial evidence for the reliability and validity of the new measure.

The initial phase ensured that the selected items were relevant and covered the physical, psychological and social aspects of oral impacts among very young children. Furthermore, the CFGs were pivotal in addressing the different conceptual and practical challenges for the development of SOHO-5. Challenges in developing OHRQoL measures for young children relate to their cognitive and emotional development [26,39] as well as the social contexts they live in [25]. Potential problems include the appropriateness and relevance of the themes covered and content of the measures, relevance of the language, readability, comprehension and ability to respond to questions appropriately, as well as the (lack of) experience of oral conditions. Furthermore, respondent burden and limited attention span are key issues when considering quality of life questionnaires at this age.

Based on the CFG data, we attempted to address some of these challenges. We used thematic analyses of the CFGs to inform the content of the SOHO-5 and included questions on all the themes that emerged from these discussions with parents, thereby ensuring that all relevant aspects of oral health related quality of life for that age were explored. Furthermore, we developed a short interviewer-administered questionnaire so that children do not lose interest or focus [26]. The questions included simple words, sentences were kept short to aid comprehension, and language accommodated the local dialect and culture. In line with evidence that 6-year-olds tend to use only the extreme and middle ratings of a 5-point scale [40], we employed 3-point answering scales, a finding confirmed by all CFGs. Moreover, following evidence from pain studies [41], we used prompt cards (with faces) to facilitate rating of oral impacts and the study identified no respondent burden issues.

The issue of time recall was also a considerable challenge. In this young age, children’s notion of time and recall periods is a difficult concept to grasp [42-44] and this was confirmed by parents in the CFG discussions. A very short time frame may have been more helpful in this respect [43], however this would not provide relevant information in terms of the impacts of oral conditions, as very few children would be expected to have experienced them, say, in the last 7 days. A more reasonable time frame would have been 3 months, but this was not feasible. Therefore, we avoided specific time recalls and opted to use the word “ever” and ask about lifetime experience of oral impacts. We do acknowledge, though, that this provides a broader picture of oral impacts and overestimates the prevalence at any given time period.

All children were able to understand and respond appropriately to the questionnaire and the vast majority did not have any difficulty comprehending the SOHO-5 questions. Actually, these questions required fewer repetitions compared to other questions, such as those about toothache experience and tooth eruption (9.1% and 9.8% respectively). The relatively more difficult concepts to comprehend were those tapping on psychosocial domains (avoiding smiling) while questions on functioning were hardly ever repeated. There may be a technical reason for this, as these were the only two questions with similar content. On a more conceptual note, this difference in comprehension between psychosocial and functional items is expected at this young age, as understanding and regulating emotions are more complex processes that require children becoming more aware of their internal world and are linked to their cognitive development later on in childhood [39,45].

Compared to OHRQoL measures for older children, the SOHO-5 questions tapped on similar broader concepts, but employed much simpler wording while there were also some notable content differences such as the question on playing and the use of two different questions on smiling. The differentiation between avoiding smiling due to toothache and due to appearance was a direct and consistent suggestion of the CFGs and may be a reflection of the relative importance of smiling for very young children. In addition to its standard role as a meaningful indicator of aesthetics and perceptions, avoiding smiling may also present a way to react to toothache which is most often a “new” and therefore unfamiliar experience at this very young age.

In terms of the psychometric properties of SOHO-5, these initial results were very satisfactory. Internal consistency reliability was established through different statistics. All inter-item correlations were positive and none was very high, and all item-total correlation coefficients were above the recommended level of 0.2 [46]. Cronbach’s alpha was 0.74, above the arbitrary threshold of 0.70 [47,48], and it was lower when any of the items was deleted. While the value of alpha tends to be higher on indices that have more questions, this study revealed very good internal consistency for the SOHO-5 despite the fact that it contains only a handful of questions.

For construct validity, we looked at the associations between SOHO-5 and different subjective oral health measures (current toothache, toothache lifetime experience, satisfaction with teeth, and presence of oral cavities), as well as with an aggregate oral health measure, based on both clinical (caries, pulp involvement) and subjective (current toothache, toothache experience) variables. All associations were significant and in the expected direction with higher SOHO-5 scores, indicating worse quality of life, for the groups reporting worse perceptions and having worse oral health. These consistent findings provide strong support for the validity of the new measure. Furthermore, clinically diagnosed active dental caries, pulp involvement and dental sepsis were associated with worse SOHO-5 scores. This indicates that dental caries may play an important role in shaping the overall perception of OHRQoL in very young children, in line with an earlier study [23]. More importantly, it practically demonstrates the ability of SOHO-5 to discriminate between different clinical groups.

Based on our results, oral diseases have considerable negative effects on children’s daily lives, as half of them reported an oral impact. This figure may actually underestimate the oral impacts on a population level, as our sample had lower prevalence of dental caries than the general child population in NHSGGC. The most prevalent impact related to difficulty eating, but other aspects of daily life, such as sleeping and smiling, were also affected. A previous study on a sample consisting also of young children has also shown that poor oral health affects smiling patterns [13]. It seems that oral diseases impact not only on the functional but also the psychosocial dimensions of life, even at a very young age.

Despite the positive initial results, the assessment of SOHO-5 should be an on-going process, by extending psychometric testing to properties not evaluated in this study, and assessing its applicability and performance in other populations. A future study, with repeated questionnaire administration, should provide information on test–retest reliability. This was not feasible in this study as we could not access the selected schools for a second visit because data collection took place very close to the end of the academic year and there was lack of available timetable spaces. Further studies should also complement the psychometric properties by looking into the responsiveness and interpretability of SOHO-5 scores. Despite being selected from the general population, our sample had considerable experience of dental caries; therefore future research should look into the discriminative properties of SOHO-5 in a healthier population sample. In the developmental phase, the study would have benefited if more parents participated in the CFGs; however, it is reassuring to note that discussions have been concordant in terms of the key themes, content, and technical characteristics of the new measure. Furthermore, involvement of children directly, rather than just their parents, in the qualitative work could also have provided further relevant information, though we acknowledge that conducting CFGs with very young children may also be open to criticism.

Developing a valid and reliable OHRQoL measure for very young children has important implications for research and practice. First, it can provide insight into the impacts of oral conditions on the daily life of young children. Dental caries is a chronic disease that can affect children from a very young age and it is important to measure its impacts on quality of life, as they may affect the psychological, social and educational development of the child [3,5,8,12]. Future research should also explore the extent to which socioeconomic and clinical factors determine subjective measures of oral health and quality of life. In addition, OHRQoL measures could be used in clinical decision making to assess the effectiveness of dental treatment, thereby advancing the pediatric outcomes research agenda [49]. Finally, SOHO-5 could also potentially be a valuable outcome for evaluating oral health promotion programs and/or service initiatives for this age group [50].

## **Conclusion**

This study has developed a new OHRQoL self-reported measure for 5-year-old children in the UK. The initial assessment of its reliability and validity showed very promising findings. Future studies should complement its psychometric testing and extend its application to patient samples as well as public health programs.

## Abbreviations

CFG: Consultation focus group; NDIP: National Dental Inspection Programme; NHSGGC: Greater Glasgow and Clyde National Health Service; OHRQoL: Oral health related quality of life; SES: Socioeconomic status; SOHO-5: Scale of Oral Health Outcomes for 5-year-old children.

## **Competing interests**

The authors declare no competing interests.

## **Authors’ contributions**

GT, RGW, YIB and LMDM conceived the study. All authors designed the study and reviewed versions of the protocol. WW and YIB organized and supervised the data collection phase of the study. HY conducted the thematic analysis of the consultation focus groups and also participated in writing the manuscript. GT analysed and interpreted the quantitative data and led the writing of the manuscript. All authors critically reviewed the manuscript and provided comments for revision. All authors read and approved the final version of the manuscript.
